# Translation and validation of the Mind-Wandering Test for Spanish adolescents

**DOI:** 10.1186/s41155-017-0066-8

**Published:** 2017-06-02

**Authors:** Carlos Salavera, Fernando Urcola-Pardo, Pablo Usán, Laurane Jarie

**Affiliations:** 1 Research Group OPIICS, University of Zaragoza, Zaragoza, Spain; 2 Faculty of Education, University of Zaragoza, c/ Pedro Cerbuna, 12, 50009 Zaragoza, Spain; 3 Faculty of Health Sciences, University of Zaragoza, c/Violante de Hungría, s/n, 50009 Zaragoza, Spain

**Keywords:** Mind wandering, Mindfulness, Validation, Adolescents, Instrumental study

## Abstract

**Background:**

Working memory capacity and fluent intelligence influence cognitive capacity as a predictive value of success. In line with this, one matter appears, that of mind wandering, which partly explains the variability in the results obtained from the subjects who do these tests. A recently developed measure to evaluate this phenomenon is the Mind-Wandering Questionnaire (MWQ).

**Objective:**

The objective of this work was to translate into Spanish the MWQ for its use with adolescents and to validate it and to analyze its relation with these values: self-esteem, dispositional mindfulness, satisfaction with life, happiness, and positive and negative affects.

**Methods:**

A sample of 543 secondary students: 270 males (49.72%) and 273 females (50.28%) were used, who completed the questionnaire, and also did tests of self-esteem, dispositional mindfulness, satisfaction with life, happiness, and positive and negative effects. The transcultural adaptation process followed these steps: translation, back translation, evaluation of translations by a panel of judges, and testing the final version.

**Results:**

Validity analyses were done of the construct (% explained variance = 52.1), and internal consistency was high (α = .766). The coefficients of correlation with the self-esteem, MASS, satisfaction with life, happiness, and affects scales confirmed the questionnaire’s validity, and a multiple regression analysis (*R* ^*2*^ = 34.1; model *F* = 24.19. *p* < 0.001) was run.

**Conclusions:**

The Spanish version of the questionnaire obtained good reliability coefficients and its factorial structure reliably replicated that obtained by the original measure. The results indicate that the Spanish version of the MWQ is a suitably valid measure to evaluate the mind-wandering phenomenon.

## Background

As human beings, we tend to be distracted by the activities we perform, which is when the mind tends to wander back to the past or to plan the future. This spontaneous tendency to produce thoughts and to freely allow our minds to wander, despite external stimuli, is considered a typical characteristic of the human mind (Smallwood and Schooler, 2006). Mind wandering is understood as a mental process during which attention is distracted from a task underway to focus on the contents that our minds intrinsically produce (Smallwood and Schooler, 2015). As it is one of the most common activities that the human mind performs, it occurs in practically all day-to-day activities, and individuals are gripped to their own mind events between 10 and 50% of the time they are awake (Kane, Brown, McVay, Silvia, Myin-Germeys & Kwapil, 2007; Killingsworth and Gilbert, 2010). Mind wandering presents wide inter-individual variability, and the mind-wandering trait appears as the personal characteristic of a tendency toward mind wandering for a given period of time (Mrazek, Smallwood, Franklin, Baird, Chin & Schooler, 2012a).

Repetitive thoughts are considered an adaptive function of human beings. Despite the negative connotations associated with this concept, mind wandering is not itself considered a negative characteristic. Similar negative connotations are attached to common terms like cognitive failures, resting state, rumination, distraction, attentional failures, absent-mindedness, repetitiveness, and the like (Baars, 2010). Planning the future is one of the most beneficial results connected with mind wandering as its appearance is associated with thoughts about the future, and not with the past or present (Schooler, Smallwood, Christoff, Handy, Reichle & Sayette, 2011). Thoughts that focus on the future are increased by self-reflection (Smallwood and O’Connor, 2011) and by prioritizing personal goals (Stawarczyk, Majerus, Maj, Van der Linden and D’Argembeau, 2011), which is reduced by negative moods (Smallwood and O’Connor, 2011). Along these lines, mind wandering comes over as an adaptive advantage as it can diminish distress by predicting future events to better adapt to one’s own environment (Bar, 2009). Mind wandering allows information that cannot be analyzed when a stimulus emerges to be systematized because the semantic manipulation of information cannot take place while a stimulus occurs (Binder, Frost, Hammeke, Bellgowan, Rao & Cox, 1999), and is thus associated with effective coping (Greenwald and Harder, 1995) and creativity (Sio and Ormerod, 2009). This anticipative capacity and planning of the future allow problems to be creatively solved (Baird, Smallwood, Mrazek, Kam, Franklin & Schooler, 2012).

High levels of mind wandering are related with low moods (Killingsworth and Gilbert, 2010) and negative thinking (Smallwood, O’Connor, Sudbery, and Obonsawin, 2007). An increase in negative thoughts in relation to mind wandering has been associated with individual levels of depression (Marchetti, Koster and De Raedt, 2012). This association may be due to mind wandering which, given the spontaneous emergence of thoughts, is associated with paying more attention to one’s own thoughts, emotions, and experiences (Smallwood and Schooler, 2015). This marked increase in self-attention may mean being at more risk of self-assessment, which has been associated with negative emotions (Mor and Winquist, 2002). The appearance of repetitive thoughts is relevant for the appearance and maintenance of emotional disorders (Aldao, Nolen-Hoeksema and Schweizer, 2010) through brooding and worrying. Although both of these constructs are related with mind wandering, they are considered to semantically differ. Indeed, worrying is defined as expecting possible negative results in the future (Borkovec, Robinson, Pruzinsky and DePree, 1983), while brooding is defined as the repetitive response model that involves the constant development of distress symptoms, and of the causes and consequences of distress (Nolen-Hoeksema, Wisco and Lyubomirsky, 2008).

Increased mind wandering and paying more attention to one’s own thoughts, emotions, and experiences have been related with low levels of self-esteem (Mrazek, et al. 2013). Nevertheless, paying more attention to oneself is not necessarily considered a negative activity for self-esteem. So mindfulness is considered a construct that contrasts with mind wandering (Mrazek, Smallwood, and Schooler, 2012b). The mindfulness construct has been defined in many forms, and all its definitions coincide in that it is a matter of paying intentionally more attention to the present time and not taking a judgemental attitude about experience (Brown and Ryan, 2003; Germer, 2005; Kabat-Zinn, 1990; Segal, Williams and Teasdale, 2002). This non-judgemental attitude makes mindfulness appear positively related with self-esteem (Kong, Wang, and Zhao, 2014; Rasmussen and Pidgeon, 2011). In turn, self-esteem is considered a predictor of satisfaction with life (Diener and Diener, 1995; Mäkikangas and Kinnunen, 2003). Hence, the aforementioned factors may be considered modulators in the relation between mind wandering and satisfaction with life.

As no valid scale exists to measure mind wandering, the usual way to assess it involves periodically interrupting individuals while they do a task, and asking them to report the extent to which their attention was related to on-task or on task-unrelated concerns (Mrazek, Smallwood, Franklin, Baird, Chin & Schooler, 2012a). In the last few years, the Mind-Wandering Questionnaire (MWQ) was developed. It is a simple validated tool designed to directly measure mind-wandering trait levels. Its design offers good reliability and validity in both adult and adolescent populations (Mrazek, 2013), and has been validated to Chinese (Luo, Zhu, Ju and You, 2016) and Japanese (Kajimura and Nomura, 2016). In Spain, studies have been conducted on mind wandering by electroencephalography in relation to movement (Melinscak, Montesano and Minguez, 2014). However, no references about psychometric studies of the construct are available. For this reason, the objective of this work was to translate into Spanish and to validate the Mind-Wandering Questionnaire and to analyze its relation with the values of self-esteem, dispositional mindfulness, satisfaction with life, happiness, and positive and negative affects among adolescents.

## Methods

### Procedure

For the Spanish MWQ adaptation purposes, the following phases were followed:

- Translation of the original scale into Spanish by a group of expert researchers in mindfulness.
- The translated scale was administered to a sample of 50 people to detect any items that did not work well, and possible difficulties in understanding because items were poorly translated or badly written. No special difficulties were found in either the items or the instrument in general.
- The work with the scale centered on the analysis, translation, and validation of the MWQ. The whole study sample (*N* = 543) was recruited from four high school centres.

### Design

**Fig. 1:**
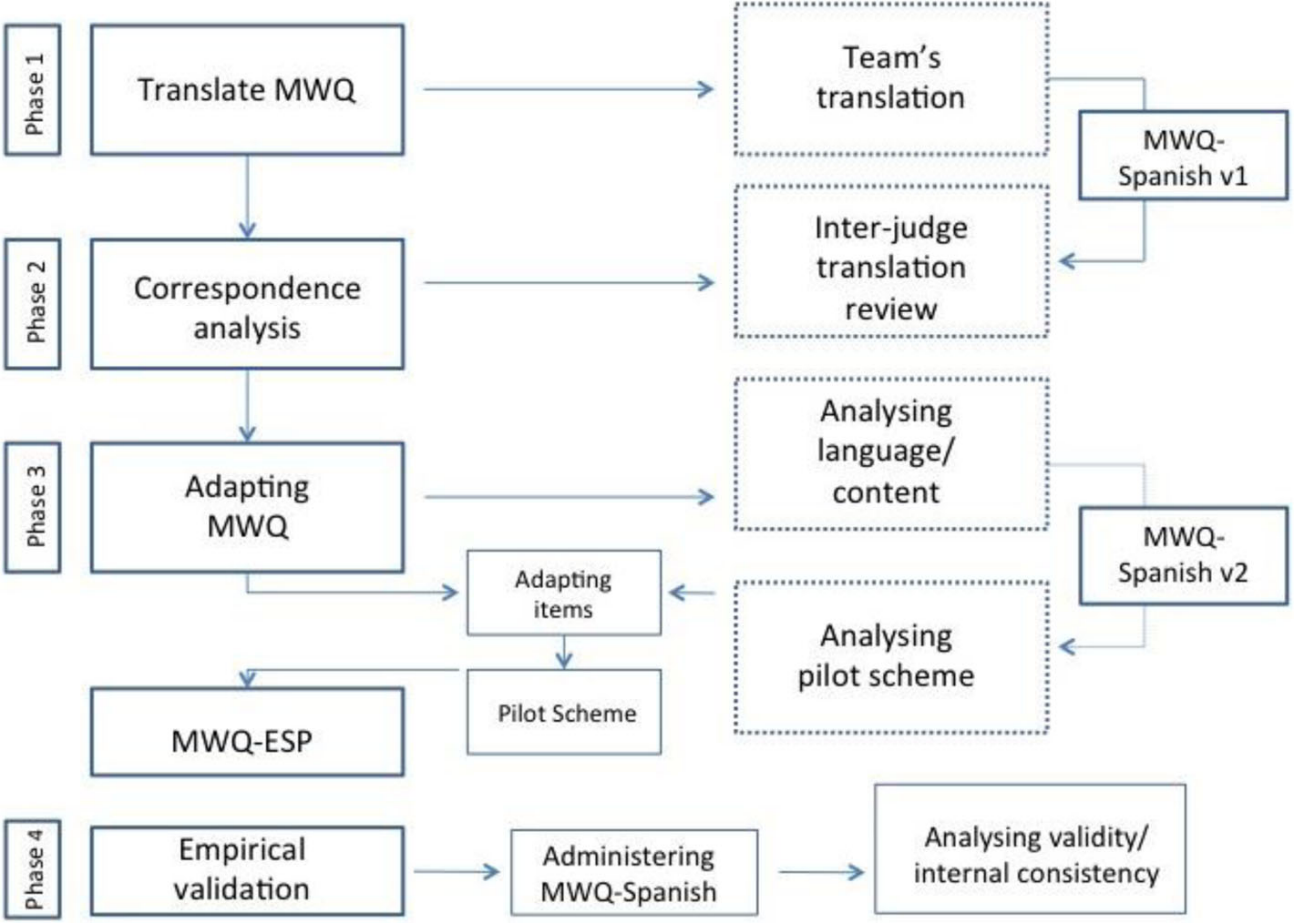
Diagram showing the phases followed to adapt the MWQ

The objective of this research was to validate the MWQ. After finishing the translation processes (Fig. 1), the first step was to study the reliability of the scales. To do this, statistics were obtained as the scale was not adapted to Spanish. This analysis informed us about the value of Cronbach’s alpha coefficient of reliability. In this questionnaire, good values were obtained (α = .766), which indicates good internal consistency among the scale elements.

### Participants

The research sample comprised 543 secondary students, 270 males (49.72%) and 273 females (50.28%). Subjects voluntarily participated and gave signed informed consents. The ethical norms of the Declaration of Helsinki were respected. The study population’s mean age was 17.24 years, and their ages ranged from 16 to 18 years, with a standard deviation of 1.015.

### Measurements

The Mind-Wandering Questionnaire (MWQ) (Mrazek, 2013), is a self-report 5-item questionnaire that evaluates the levels of the mind-wandering trait. It is a 6-point Likert-type scale that goes from 1 (almost never) to 6 (almost always). Some item examples are “I have difficulty maintaining focus on simple or repetitive work” or “I do things without paying full attention”. The total MWQ score is the sum of the five items within a 5–30 range. After obtaining permission from the author of the MWQ, it was translated. The results and its reliability/validity are described in later sections of this document.

The Mindful Attention Awareness Scale (MAAS) (Brown and Ryan, 2003) is a simple scale that is quickly administered and globally evaluates an individual’s dispositional capacity of being alert and aware of the present experience in his/her daily life. MAAS is a 15-item questionnaire that scores on a Likert scale from 1 (almost always) to 6 (almost never). It measures the frequency of the mindfulness state in activities of daily living without having to train subjects. Scores are obtained using the arithmetic mean of all the items, and high scores indicate a greater mindfulness state. In the present study, this scale shows high internal consistency with a Cronbach’s alpha coefficient of 0.878.

The Subjective Happiness Scale (Lyubomirsky and Lepper, 1999) is an overall measure of subjective happiness that evaluates a molar category of well-being as an overall psychological phenomenon by considering the definition of happiness from the respondent’s perspective. It comprises four items with Likert-type responses and is corrected by summing the points obtained and then dividing them by the total number of items. In the present study, this scale shows high internal consistency with a Cronbach’s alpha coefficient of 0.845.

The Satisfaction with Life Scale (Diener, Emmons, Larsen and Griffin, 1985) is a 5-item scale that evaluates satisfaction with life. The participants must indicate the extent to which they agree with each statement on a 7-point Likert scale (from 1 = I strongly disagree to 7 = I strongly agree). Scores may range from 5 to 35 points; higher scores indicate greater satisfaction with life. This scale in this study shows high internal consistency with a Cronbach’s alpha coefficient of 0.863.

Rosenberg’s Self-esteem Scale (Rosenberg, 1965) is self-applied and contains 10 statements of the feelings that each person feels about him/herself; five in the positive sense (items 1, 2, 4, 6, and 7) and five in the negative sense (items 3, 5, 8, 9, and 10). It is a Likert-type scale whose theoretical values fluctuate between 10 (low self-esteem) and 40 (high self-esteem). The Cronbach’s alpha obtained by this scale is 0.876.

The PANAS schedule (Watson, Clark and Tellegen, 1988), this being the positive and negative affect schedule (PANAS), includes 20 items, of which 10 refer to positive affects (PA) and 10 to negative affects (NA) on two Likert-type scales. They all refer to the time the scale is answered (right now), with a score from 0 (not at all emotional) to 5 (extremely emotional). This scale shows an alpha of 0.790 for PA and one of 0.874 for NA.

### Data analysis

The statistical analysis was done using version 22.0 of the SPSS software package for Windows. Factorial analyses were done. By reducing data, this technique is used to explain the variability among observed variables in terms of a smaller number of non-observed variables called factors. The observed variables were modeled as linear combinations of factors, plus error expressions. The intention was to analyze the consistency of the scale factors. In this study, a combination of EFA and CFA was performed. The majority of the studies chose the use of EFA for factor analysis. Others used CFA, for specific hypothesized factor structure proposed in EFA. DeVellis (2003) suggested the combined use of EFA and CFA for more consistent results on the psychometric indices of new scales. Recently, this suggestion of considering the combined use of EFA and CFA during the evaluation of construct validity of new measures has been approved by other authors, in order to provide more consistent psychometric results (Morgado, Meireles, Neves, Amaral and Ferreira, 2017).

Confirmatory analyses were run with the AMOS program, v24.0, with the study sample to verify if the factorial structure of the Spanish version matched that in the original version. Following the recommendations by Batista and Coenders (2000), the maximum likelihood estimation method was used rather than the weighted least squares method given the small sample size and few variables involved. As variables were measured at the ordinal level, estimations were made with polychoric correlations matrices instead of with covariance matrices.

## Results

### Construct validity

Construct validity was firstly analyzed. Although it is not a factor analysis technique, it was used to factorize the principal components analysis with varimax rotation as it can serve as an exploratory tool. After checking the validity of the factorial analysis with the following criteria: the correlations matrix had a large number of correlations (87.4%) with a value over 0.30, and a determining factor that equaled 0.001. The result of Bartlett’s sphericity test showed that the variables were not independent (Bartlett’s test = 321.43, *p* < .001). The obtained Kaiser-Meyer-Olkin (KMO) test value for sampling sample adequacy was 0.788. This indicated that the correlations between pairs of variables can be explained by the other variables. All the measures of sampling adequacy (MSA) values were over .78. These values indicated that running a factorial analysis of the correlations matrix was adequate. As Table 1 shows, a factor was obtained with an eigenvalue higher than 1 by assigning the factor an item as a criterion in that which presents a factorial load over 0.40, which explained 52.1% of total variance.

**Table 1:** Exploratory factorial analysis

	Factor 1 MWQ
Item 1	0.631
Item 2	0.74
Item 3	0.783
Item 4	0.771
Item 5	0.672
Eigenvalues	2.6
% explained variance	52.1

**Fig. 2:**
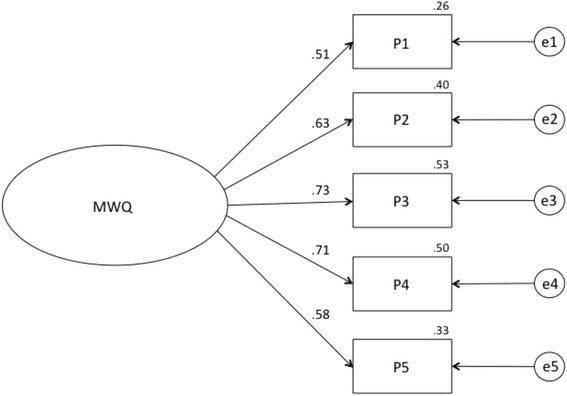
Estimated normalized parameters of the CFA model

Figure 2 offers the confirmatory factor analysis (CFA) result of the model generated in the exploratory study, along with the structural equations from the method that obtained the maximum likelihood. This confirmed that the model was adequate because a sustainable model was obtained, which comprised a total of one factor and five indicators (Fig. 2). The normalized regression coefficients were statistically significant (*p* < 0.05), with values above 0.5, which indicates that all the indicators satisfactorily saturated with the latent variable.

The different fit indices were suitable for the model’s fit. Thus, we can state that the model proposed for the factorial scale structure is sustainable: χ^2^(5) = 18.3; *p* = 0.003; χ^2^/gl = 3.7; *GFI* = 0.94; *AGFI* = 0.96; *CFI* = 0.96; *NFI* = 0.94; *TLI* = 0.93; *RMSEA* = 0.07. 90% CI (0.05–0.11).

### Internal consistency

It allowed us to estimate the reliability of the measuring instrument with a set of items expected to measure the same construct or the theoretical dimension. The scale’s Cronbach’s alpha was higher than 0.75, so it was assumed that the items which comprised the scale measured the same construct and correlated well (Table 2).

**Table 2:** The internal consistency of the MWQ

Items	Mean	SD	Item-test correlation	Alpha if eliminated	Scale
MWQ					
Item 1	2.58	1.23	.650**	0.755	Mean = 2.75 Alpha = 0.766
Item 2	2.66	1.26	.742**	0.713	
Item 3	2.53	1.15	.758**	0.700	
Item 4	3.06	1.25	.760**	0.702	
Item 5	2.92	1.29	.688**	0.744	

*p < 0.05; **p < 0.01

### Convergent validity

We also analyzed convergent validity with the other constructs analyzed in this work to test that the constructs expected to be related were indeed related (Table 3).

**Table 3:** Convergent validity

	Total sample	Males	Females
Self-esteem	−.326**	−.365*	−.323**
MAAS	−.495**	−.448**	−.503**
Satisfaction with life	−.296**	−.300*	−.294**
Happiness	−.305**	−.299**	−.320**
Positive affects	−.336**	−.344*	−.337**
Negative affects	.413**	.483**	.402**

*p < 0.05; **p < 0.01

### Multiple regression analysis

Finally, a multiple regression analysis was performed (Table 4) to analyze the possible relation between the independent variables (self-esteem, MAAS, satisfaction with life, PA, and NA) that act as predictors or explanatory variables, and another independent variable, the MWQ.

**Table 4:** Predictors of the MWQ

Predictors	B (ET)	Beta	*t*
Self-esteem	0.01 (0.05)	−0.01	−0.07
MAAS	−2.00 (0.28)	−0.38	−7.11***
Satisfaction with life	−0.02 (0.26)	−0.01	−0.08
Happiness	0.04 (0.08)	0.04	0.52
Positive affects	−0.15 (0.04)	−0.21	−3.52**
Negative affects	0.14 (0.04)	0.26	4.13***
Adjusted *R* ^2^ (%)	34.1		
Model	F(6543) = 24.19. *p* < 0.001		

*p < 0.05; **p < 0.01; ***p < 0.01

The MAAS and NA had a significant and negative effect with the MWQ as high values for these variables were associated with low MWQ values. PA had a significant direct effect with the MWQ and the high values of these affects were associated with high MWQ values.

## Discussion

The objective of the present study was to translate into Spanish and analyze the psychometric properties of the Mind-Wandering Questionnaire (MWQ) with adolescents. Studies into this construct conducted with adolescent populations are much scarcer than those done with adult populations. Some research works have evaluated mind wandering in adolescent samples by identifying relevant indicators (Luo, Zhu, Ju, and You, 2016; Mrazek et al. 2013). The results revealed that the Spanish version of the MWQ for adolescents evidences validity and reliability.

Regarding evidence for construct validity, a correlations analysis was firstly done with the five-scale items. The results showed some positive and significant results among them, with values above those obtained by Mrazek in 2013. These scores can be accounted for by the homogeneity that occurs in the scores of the variables that make up subjective well-being. Indeed, this situation has led several authors to consider the possible existence of some higher-order construct that covers several of what we now often consider to be synonyms, measured by different scales (e.g., subjective well-being, personal well-being, satisfaction with life, or happiness), which have shown significantly positive and generally high correlations with one another, and apparently overlap. Although these variables have clearly different characteristics, it is generally considered that their respective overall values are equally good indicators of subjective well-being. However, the observed correlations have not been high enough to be able to state that they measure identical constructs (Banati and Diers, 2016; Casas, Baltatescu, Bertrán, González, and Hatos, 2013; Diener, et al. 1999; Nilsen and Bacso, 2017). The scores obtained with the MWQ would indicate that somehow this new construct could form part of subjective well-being.

Secondly, the factorial structure of the MWQ was analyzed by a confirmatory factorial analysis. The results indicated good data fit, which corroborated the scale’s dimensional structure, and also coincided with both the initial questionnaire postulates (Mrazek et al.*,* 2013) and the factorial structure obtained in the original questionnaire version. The values obtained for scale reliability through Cronbach’s alpha were acceptable in all the items and were similar to those found in not only the original version, but also in subsequent studies conducted in different contexts (Kajimura and Nomura, 2016; Luo et al. 2016). This could be an indication of the appropriateness of using this scale with an adolescent population. To examine the scale’s concurrent validity, the structural equations model was tested, in which it was hypothesized that self-esteem, satisfaction with life, subjective happiness, positive/negative affects, and dispositional mindfulness predicted the results of the mind-wandering phenomenon. Here, gender differences were found as the results for the female participants in the MWQ scale obtained higher correlation indices with the MAAS Scale and PA, while the males’ results were higher for self-esteem and NA. This gender discrepancy in the affects themes has already been pointed out by some authors (Salavera, Usán, Antoñanzas, Teruel and Lucha, 2017). The multiple regression results showed that happiness, self-esteem, and satisfaction with life did not seem to influence the mind-wandering phenomenon. These three constructs have a lot to do with a person’s disposition and with the subjective evaluation of his or her well-being. So up to a point, it would be logical to understand that with a phenomenon like mind wandering, the variables that require greater awareness about the subject’s conscience state do not act as predictors, which was the case of the present research. Only PA had a significant and positive effect with the MWQ as high values for PA were associated with high MWQ values. There is an explanation for this as positive affect includes mood states and various emotions with pleasant, almost agreeable, subjective content, and with conditions or events that positively inform about how life is going (Luna, 2012), which falls in line with mind wandering. In the same way, dispositional mindfulness and NA predicted a negative and significant effect with the MWQ as high values for these variables were associated with low MWQ scores, which indicates that despite an increase in emotional regulation skills taking place in adolescence, an increase in negative affect states has also been detected during this life period (Larson, Moneta, Richards and Wilson, 2002). Thus the self-assessments that adolescents make can activate negative emotions, like fear, sadness, or rejection, which would explain why the mind-wandering process correlates inversely with negative affects.

We should, however, point out that the present study has some limitations. Firstly, the evidence found for validity and reliability must be considered provisional as our sample size, especially males, was small. Future studies should verify gender effects with a larger study sample to evaluate the relation of these constructs over the years. Secondly, it would be necessary to test the instrument’s factorial structure in different contexts. As future research lines, and like other works (Diener, Suh, Lucas and Smith, 1999; Hampel and Petermann, 2006; Mrazek, et al., 2012b), we should make an in-depth examination of the interaction among mind wandering, psychological factors, and different life events, and continue to investigate the relation of mind wandering with different subjective well-being components (subjective happiness, self-esteem, and satisfaction with life and affects), and consider programs that promote the use of active strategies to enhance personal well-being in adolescents.

## Conclusions

To conclude, our results revealed that the Spanish version of the MWQ for adolescents offers preliminary evidence for validity and reliability, and along the same lines as the results obtained in the original version. In addition, this questionnaire could be useful for indirect measurement of the effectiveness of interventions with mindfulness. The inverse relationship found with the MAAS questionnaire (which measures mindfulness-trait) leads us to think that it can serve as an indicator of mind-wandering moments, which will decrease as the practice of mindfulness advances. Therefore, the Spanish version for adolescents may be considered a preliminary adaptation of the original questionnaire version, and the results justify its use for evaluating the mind-wandering phenomenon in the Spanish adolescent population. The present research results encourage us to continue seeking new questions to help us define new tools and methodologies and to find some answers to make progress in building mindfulness practices in adolescents.

## Appendix 1

Mind-Wandering Scale (Mrazek, 2013)

Cuestionario de vagabundeo de la mente.

Translation Spanish (Salavera, Urcola-Pardo, Usán and Jarie)

1. Me resulta difícil mantener la concentración en trabajos sencillos o repetitivos
2. Mientras leo, me doy cuenta de que no he estado pensando en el texto y que, por lo tanto, tengo que leerlo otra vez
3. Hago las cosas sin prestar total atención
4. Me encuentro escuchando a medias al mismo tiempo que estoy pensando en otra cosa
5. Me distraigo durante conferencias y presentaciones

## References

[CR1] Aldao A, Nolen-Hoeksema S, Schweizer S (2010). Emotion-regulation strategies across psychopathology: a meta-analytic review. Clinical Psychology Review.

[CR2] Baars BJ (2010). Spontaneous repetitive thoughts can be adaptive: postscript on “Mind Wandering”. Psychological Bulletin.

[CR3] Baird B, Smallwood J, Mrazek MD, Kam JW, Franklin MS, Schooler JW (2012). Inspired by distraction: mind wandering facilitates creative incubation. Psychological Science.

[CR4] Banati P, Diers J (2016). Measuring adolescent well-being: National Adolescent Assessment Cards (NAACs). Innocenti Research Brief.

[CR5] Bar M (2009). The proactive brain: memory for predictions. Philosophical Transactions of the Royal Society B-Biological Sciences.

[CR6] Batista JM, Coenders G (2000). Modelos de Ecuaciones Estructurales (Structural Equation Modeling).

[CR7] Binder JR, Frost JA, Hammeke TA, Bellgowan PSF, Rao SM, Cox RW (1999). Conceptual processing during the conscious resting state: a functional MRI study. Journal of Cognitive Neuroscience.

[CR8] Borkovec TD, Robinson E, Pruzinsky T, DePree JA (1983). Preliminary exploration of worry: some characteristics and processes. Behaviour Research and Therapy.

[CR9] Brown KW, Ryan RM (2003). The benefits of being present. Mindfulness and its role in psychological well-being. Journal of Personality and Social Psychology.

[CR10] Casas F, Baltatescu S, Bertrán I, González M, Hatos A (2013). School satisfaction among adolescents: testing different indicators for its measurement and its relationship with overall life satisfaction and subjective well-being in Romania and Spain. Social Indicators Research.

[CR11] DeVellis RF (2003). Scale development: theory and applications.

[CR12] Diener E, Diener M (1995). Cross-cultural correlates of life satisfaction and self-esteem. Journal of Personality and Social Psychology.

[CR13] Diener, E., Emmons, R.A., Larsen, R.J. & Griffin, S. (1985). The Satisfaction with Life Scale. Journal of Personality Assessment, *49*, 71–75. 10.1207/s15327752jpa4901_13 16367493

[CR14] Diener E, Suh E, Lucas R, Smith H (1999). Subjetive well-being: three decades of progress. Psychological Bulletin.

[CR15] Hampel, P. & Petermann, F. (2006). Perceived stress, coping, and adjustment in adolescents. Journal of Adolescent Health, *38* (4), 409–415. 10.1016/j.jadohealth.2005.02.014. 16549302

[CR16] Germer CK, Germer CK, Siegel RD, Fulton PR (2005). Mindfulness: what is it? What does it matter?. Mindfulness and psychotherapy.

[CR17] Greenwald DF, Harder DW (1995). Sustaining fantasies, daydreams, and psychopathology. Journal of Clinical Psychology.

[CR18] Kabat-Zinn J (1990). Full catastrophy living. How to cope with stress, pain and illness using mindfulness meditation.

[CR19] Kajimura S, Nomura M (2016). Development of Japanese versions of the Daydream Frequency Scale and the Mind Wandering Questionnaire. Shinrigaku Kenkyu.

[CR20] Kane MJ, Brown LH, McVay JC, Silvia PJ, Myin-Germeys I, Kwapil TR (2007). For whom the mind wanders, and when: an experience-sampling study of working memory and executive control in daily life. Psychological Science.

[CR21] Killingsworth MA, Gilbert DT (2010). A wandering mind is an unhappy mind. Science.

[CR22] Kong F, Wang X, Zhao J (2014). Dispositional mindfulness and life satisfaction: the role of core self-evaluations. Personality and Individual Differences.

[CR23] Larson RW, Moneta G, Richards MH, Wilson S (2002). Continuity, stability, and change in daily emotional experience across adolescence. Child Development.

[CR24] Luna FJ (2012). Bienestar subjetivo y satisfacción escolar en la adolescencia. Tesis doctoral.

[CR25] Luo Y, Zhu R, Ju E, You X (2016). Validation of the Chinese version of the Mind-Wandering Questionnaire (MWQ) and the mediating role of self-esteem in the relationship between mind-wandering and life satisfaction for adolescents. Personality and Individual Differences.

[CR26] Lyubomirsky, S. & Lepper, H. (1999). A measure of subjective happiness: preliminary reliability and construct validation. Social Indicators Research, *46*, 137–155.

[CR27] Mäkikangas A, Kinnunen U (2003). Psychosocial work stressors and well-being: self-esteem and optimism as moderators in a one-year longitudinal sample. Personality and Individual Differences.

[CR28] Marchetti I, Koster EH, De Raedt R (2012). Mindwandering heightens the accessibility of negative relative to positive thought. Consciousness and Cognition.

[CR29] Melinscak F, Montesano L, Minguez J (2014). Discriminating between attention and mind wandering during movement using EEG. Proceedings of the 6th International Brain–Computer Interface Conference.

[CR30] Mor N, Winquist J (2002). Self-focused attention and negative affect: a meta-analysis. Psychological Bulletin.

[CR31] Morgado, F. F. R., Meireles, J. F. F., Neves, C. M., Amaral, A. C. S., & Ferreira, M. E. C. (2017). Scale development: ten main limitations and recommendations to improve future research practices. *Psicologia: Reflexão e Crítica, 30*(3). 10.1186/s41155-016-0057-1 PMC6966966 32025957

[CR32] Mrazek MD, Phillips DT, Franklin MS, Broadway JM, Schooler JW (2013). Young and restless: validation of the Mind-Wandering Questionnaire (MWQ) reveals disruptive impact of mind-wandering for youth. Frontiers in Psychology.

[CR33] Mrazek MD, Smallwood J, Franklin MS, Baird B, Chin J, Schooler JW (2012). The role of mind-wandering in measurements of general aptitude. Journal of Experimental Psychology: General.

[CR34] Mrazek MD, Smallwood J, Schooler JW (2012). Mindfulness and mind-wandering: finding convergence through opposing constructs. Emotion.

[CR35] Nilsen ES, Bacso SA (2017). Cognitive and behavioural predictors of adolescents’ communicative perspective-taking and social relationships. Journal of Adolescence.

[CR36] Nolen-Hoeksema S, Wisco BE, Lyubomirsky S (2008). Rethinking rumination. Perspectives on Psychological Science.

[CR37] Rasmussen MK, Pidgeon AM (2011). The direct and indirect benefits of dispositional mindfulness on self-esteem and social anxiety. Anxiety, Stress and Coping.

[CR38] Rosenberg, M. (1965). Society and the Adolescent Self-Image. Princeton, New Jersey: Princeton University Press.

[CR39] Salavera, C., Usán, P., Antoñanzas, J. L., Teruel, P., & Lucha, O. (2017). Affects and personality: a study students. *Annales Medico-Psychologiques* (in press). 10.1016/j.amp.2016.06.014

[CR40] Schooler JW, Smallwood J, Christoff K, Handy TC, Reichle ED, Sayette MA (2011). Meta-awareness, perceptual decoupling and the wandering mind. Trends in Cognitive Sciences.

[CR41] Segal ZV, Williams JM, Teasdale JD (2002). Mindfulness-based cognitive therapy for depression: a new approach to preventing relapse.

[CR42] Sio UN, Ormerod TC (2009). Does incubation enhance problem solving? A meta-analytic review. Psychological Bulletin.

[CR43] Smallwood J, O’Connor RC (2011). Imprisoned by the past: unhappy moods lead to a retrospective bias to mind wandering. Cognition & Emotion.

[CR44] Smallwood, J., O’Connor, R. C., Sudbery, M. V., & Obonsawin, M. (2007). Mind-wandering and dysphoria. *Cognition and Emotion, 21*(4), 816–842. 10.1080/02699930600911531.

[CR45] Smallwood, J., & Schooler, J. W. (2006). The restless mind. *Psychological Bulletin, 132*(6), 946–958. 10.1037/0033-2909.132.6.946. 17073528

[CR46] Smallwood J, Schooler JW (2015). The science of mind wandering: empirically navigating the stream of consciousness. Annual Review of Psychology.

[CR47] Stawarczyk D, Majerus S, Maj M, Van der Linden M, D’Argembeau A (2011). Mind-wandering: phenomenology and function as assessed with a novel experience sampling method. Acta Psychologica.

[CR48] Watson, D., Clark, L.A. & Tellegen, A. (1988). Development and validation of brief measures of positive and negative affect: the PANAS scales. Journal of Personality and Social Psychology, *54*, 1063–1070. 10.1037//0022-3514.54.6.1063 3397865

